# Estimation of Density-Dependent Mortality of Juvenile Bivalves in the Wadden Sea

**DOI:** 10.1371/journal.pone.0102491

**Published:** 2014-08-08

**Authors:** Henrike Andresen, Matthias Strasser, Jaap van der Meer

**Affiliations:** 1 Department of Marine Ecology, Royal Netherlands Institute for Sea Research, Den Burg, The Netherlands; 2 Division Coastal Ecology, Alfred Wegener Institute - Wadden Sea Station Sylt, List, Germany; 3 Department of Theoretical Biology, Vrije Universiteit, Amsterdam, The Netherlands; McGill University, Canada

## Abstract

We investigated density-dependent mortality within the early months of life of the bivalves *Macoma balthica* (Baltic tellin) and *Cerastoderma edule* (common cockle) in the Wadden Sea. Mortality is thought to be density-dependent in juvenile bivalves, because there is no proportional relationship between the size of the reproductive adult stocks and the numbers of recruits for both species. It is not known however, when exactly density dependence in the pre-recruitment phase occurs and how prevalent it is. The magnitude of recruitment determines year class strength in bivalves. Thus, understanding pre-recruit mortality will improve the understanding of population dynamics. We analyzed count data from three years of temporal sampling during the first months after bivalve settlement at ten transects in the Sylt-Rømø-Bay in the northern German Wadden Sea. Analyses of density dependence are sensitive to bias through measurement error. Measurement error was estimated by bootstrapping, and residual deviances were adjusted by adding process error. With simulations the effect of these two types of error on the estimate of the density-dependent mortality coefficient was investigated. In three out of eight time intervals density dependence was detected for *M. balthica*, and in zero out of six time intervals for *C. edule*. Biological or environmental stochastic processes dominated over density dependence at the investigated scale.

## Introduction

Survival during the early life phase plays a central role in population dynamics of marine invertebrates [1]. Many marine species produce an excess of larvae, which prepares the population for unpredictable events [2]. Broadcast spawning by bivalves can create a wide range of larval densities. When comparing between years, the number of offspring does not increase proportionally with increasing stock of reproductive animals [3]. Early juvenile mortality typically leads to asymptotic stock-recruitment curves. This shape of the relationship points towards density-dependent mortality in the pre-recruit phase. Recruitment is the subjectively defined stage at which juvenile survivors are regarded as added to a population. In our study system in the Wadden Sea, juvenile bivalves are usually termed recruits at sampling in August (e.g. [4]). Pre-recruit and early post-recruit survival of Wadden Sea bivalves is indeed higher in years with low densities [5]–[7]. The recruitment period is the key to marine population dynamics, as it largely sets year class strength. Still, we do not know much about the causes of density-dependent mortality in this early life phase, how prevalent it is in the first place, and when exactly mortality is density-dependent during this critical period of the life-history.

**Figure 1:**
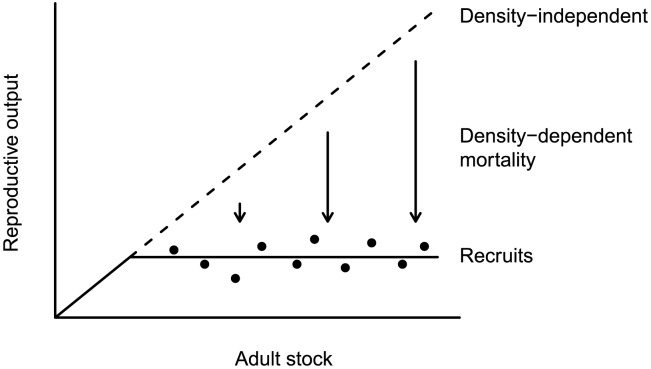
Scheme of an asymptotic adult-recruit relationship. Reproductive output of the adult stock is proportional to the stock size, but through density-dependent mortality during the pre-recruit phase, a stock-recruitment relationship is lacking (after [12]).

Mechanisms that could lead to density-dependent mortality are competition, infections, and risky density-dependent behavior such as migration [8], [9]. Density-independent mortality can be age related or have extrinsic causes such as weather events or food supply. Mortality through predation can have both density-dependent [10], [11] and -independent aspects. If pre-recruit mortality is density-independent, the mortality rate is constant over all densities, and the more eggs are produced, the more recruits will be there. If pre-recruit mortality is density-dependent, it is higher at high densities. A higher reproductive output means proportionally fewer survivors (Fig. 1).

The bivalve species investigated in this study are the Baltic tellin *Macoma balthica* (L.) and the common cockle *Cerastoderma edule* (L.). Both are abundant infaunal bivalve species in the Wadden Sea. They spawn large quantities in spring or early summer, and after a planktonic larval phase of several weeks, the juveniles settle to the soft sediment. *C. edule* has a hard shell and is a suspension feeder, while *M. balthica* buries deep to avoid predators, it is predominantly a deposit feeder (facultative suspension feeder) [14]. *C. edule* densities show stronger variability between years [3], and their spatial distribution is more patchy [13] compared to *M. balthica*. *M. balthica* is less variable in occurrence in time and space, suggesting a stronger role of density dependence.

We analyzed a data set that addressed the first half year of life of the bivalves, from settlement to recruitment. The young bivalves were sampled in ten transects four to five times during spring and summer of three years. Density dependence across space was assessed for separate time periods. Our main aim was to determine whether mortality was spatially density-dependent.

## Materials and Methods

### Ethics Statement

Field sampling was allowed with a permit from the Schleswig-Holstein Wadden Sea National Park administration to the Wadden Sea Station Sylt.

### Theoretic background

Beverton and Iles [15] present a procedure for analyzing density dependence using a regression between pairs of densities of successive sampling events. It assumes mortality according to the following equation:
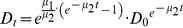
(1)where *D_t_* is the density at any time *t*, *D*_0_ is the initial density at *t* = 0, *μ*_1_ is the density-independent mortality coefficient and *μ*_2_ is the density-dependent coefficient. Then in the regression of the densities at the end (log*D_t_*_+1_) on the corresponding densities at the beginning (log*D_t_*) of a specific time interval, the slope is 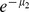 and thus the density-dependent component of mortality can be estimated. The slope of the regression should be <1 to diagnose density dependence. Stronger deviation from a slope of 1 means stronger density dependence. However, in the presence of measurement error, a simple linear regression of log*D_t_*_+1_ on log*D_t_* would yield estimates of slopes that are biased downward (and hence of *μ*_2_ that are too large), exaggerating a departure from the null hypothesis of no density dependence. To solve this without quantitative knowledge of the measurement error, one could fit a density-independent model and then ask retrospectively how much census error would have been required to produce the observed levels of density dependence [16]. If much error would have been required, the conclusion of density-dependent mortality may be regarded as robust. In another approach, called SIMEX (Simulation-extrapolation) [17], a method that needs an error model, additional observation error is put into the data. From the resulting parameters it is then extrapolated back to predict what happens under reduced observation error. These two approaches inspired our method. We compared the observed apparent level of density dependence with the results of simulations for a range of given strengths of density dependence. The simulations incorporated the effects of measurement error and process error, i.e. actual biological variability as opposed to sampling variation [18], [19], in two separate steps.

### Data

The study was conducted in the Sylt-Rømø Bight, a tidal basin in the northern Wadden Sea (German Bight). The tidal range in the bight is about 2 m; a third of the basin consists of intertidal flats. Samples of post-settlement bivalves were collected in the intertidal. Ten transects were placed perpendicular to the coast along 20 km of shoreline. There were three stations on each transect, one site in the upper intertidal, one in the middle and one in the low intertidal. Every station on the transect was sampled with four replicates. Sampling took place four to five times per year in regular intervals of about six weeks, starting in spring and ending in autumn, in the years 1996 to 1998. A sampling round covering all ten transects took usually about one week. The area of the corers used was increased during the year to adjust sampling effort to declining individual densities (Table 1). Samples were fixed in buffered 5% formalin in seawater. Before sorting and counting they were sieved through a 0.125 mm screen. By the time of the last sampling round each year most juvenile bivalves were bigger than 1.5 mm and the samples were sieved in the field through a 1.0 mm mesh. For details see [20]. The abundances were summarized per transect and not per site, because *Macoma balthica* and *Cerastoderma edule* can migrate to the higher intertidal during summer, which could give the impression of density dependence but would be due to a habitat shift. One missing value was substituted with the average of the other three replicates. In 1996 the mid intertidal was not sampled in one transect, here the values were not imputed but the transect was left out for that year. Originally this data collection was part of a study on settlement timing and recruitment in relation to winter temperature [20], but the sampling design makes it suitable to analyze the data for density-dependent mortality as well. There are no dissimilar trends in recruitment in recent years in this region of the Wadden Sea (M. Strasser unpubl. data). In this analysis only data from the observed settlement peak onwards were included. In *M. balthica* there were eight periods beyond the settlement peaks in the three years; for the later arriving *C. edule*, six periods of density decline could be analyzed.

**Table 1:** Sampling time scheme with corer areas (cm^2^).

Year	Apr	May	Jun	Jul	Aug	Sep	Oct
1996		5	10		10		284
1997	10	10	10		20	284	
1998		10		20	20		284

### Data analysis

#### Accounting for measurement error only

**Figure 2:**
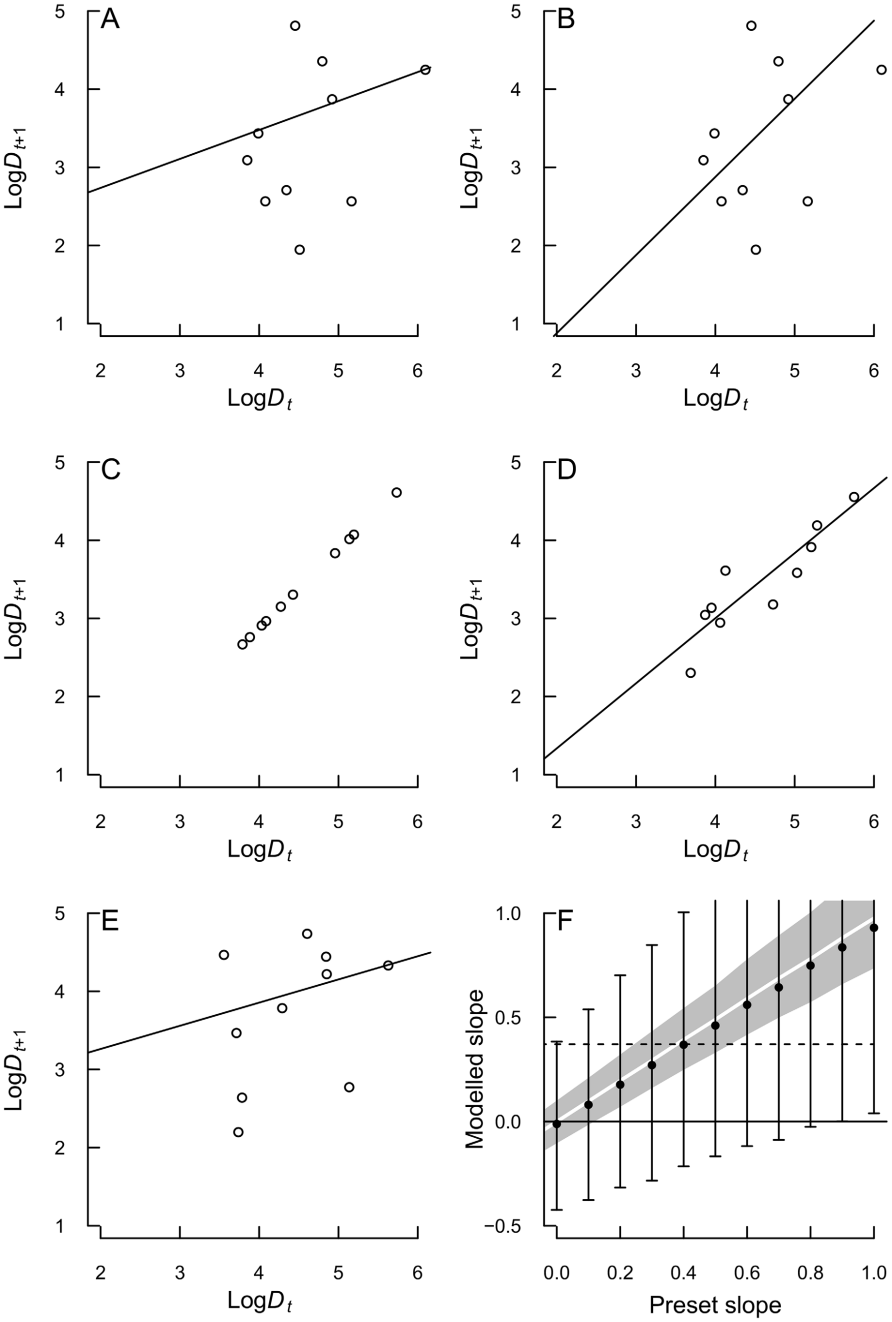
Illustration of the steps within the analysis of density dependence. A: regression through observed data of *Macoma balthica* for the time interval May to June 1997. B: preset slope forced through observed data (in this example slope = 1 for density independence). C: deterministic data calculated from observed data to lie on hypothetical regression line from previous step. D: regression after measurement error, estimated by bootstrapping, was added to deterministic data, one example of 10000 simulations. E: additionally, before adding measurement error, process error was added to log*D_t_*_+1_, to arrive at observed residual deviance from first step, one example of 10000 simulations. F: modelled slope values with prediction intervals for various preset slopes, simulated with observed measurement error alone (white line for slope values and grey bootstrap interval) and observed process error additionally (black dots and whiskers). The dashed line gives the observed slope from step A. In this case the observed slope falls within the modelled prediction interval, whatever the preset slope (i.e. assumed true slope) is.

In the data analysis procedure, first, a linear regression was fit of the log counts+1 in the ten transects at the end of a time step on the log counts+1 at the beginning of the time interval (example Fig. 2A). The regression was done as a GLM (Generalized linear model) with the quasipoisson family to allow for overdispersion, while the associated log link suits the exponential nature of mortality and implicates that density dependence is modeled as a function of log density. Sampling areas were changed over time and this was accounted for by using an offset, namely the log of the ratio of the sampling areas at the end and at the beginning of a time interval. For both species a regression model was fitted for every period. The observed slope was subsequently compared to the outcomes of simulations. The statistical question to be answered is, which hypothetical true slopes may lead to the observed slope under the observed measurement error? For this, a density-independent model (slope = 1) and models with different predefined levels of density dependence (slopes<1) were forced through the observed data (example Fig. 2B). The preset slopes and the respective resulting intercepts were then used to calculate deterministic data without error (Fig. 2C) from the observed data. Next, error was added by generating random deviates with the observed measurement error (details on quantification of measurement error below). Through these simulated data, a regression was fit again (example Fig. 2D). Adding the error and fitting the regression was repeated 10000 times per preset slope, and the average resulting slopes with 95% prediction intervals (white line and grey area in example Fig. 2F) plotted against the preset slopes to compare it with the slope calculated from the observed field data (dashed line in Fig. 2F).

The measurement error to be added to the data was estimated for each date separately with bootstrapping: the counts in the four cores for each of the three sites per transect were sampled with replacement, and the drawings of the three sites per transect were summed. This was repeated 100000 times and the variance of the sum per transect was calculated. For each species, per time step one dispersion parameter was used for all transects for the generation of stochastic data; it was modeled as variance = mean*^b^*, which is known as Taylor's power law [21]. The exponent *b* had values around 1, pointing to a Poisson distribution, though it was significantly >1 (*p*<0.05) in 6 out of 20 cases (time steps × species). To be conservative, as the effect of measurement error is central to this study, when the exponent was >1, whether significant or not, the variance calculated with the actual value of *b* was used for generating stochastic data with the negative binomial distribution. When *b* was ≤1, Poisson distributed values were generated.

#### Incorporating process error

The residual deviance in the simulations with measurement error was much lower than in the original regression (compare Fig. 2A and D). The measurement error was estimated from the data by bootstrapping, so the remaining error must be process error, due to environmental variation and biological processes. This was included in an extra step before incorporating measurement error. Lognormal errors were added to the abundance at the end of a time interval. By that, process error is treated as an additional source of density-independent fluctuation [22]. In case the simulated abundance after adding process error was <1, it was set to 0 (otherwise the dispersions parameter of the negative binomial distribution is not defined). For each slope, we found the amount of process error that, together with the subsequently added measurement error, on average resulted in a regression with the original residual deviance (±2%). In the same way as for the measurement error, the average resulting slopes (example Fig. 2E) and the enlarged confidence intervals were plotted against the preset, true slopes (example Fig. 2F). If, at the preset slope 1 for density independence, the lower end of the confidence interval of the modelled slope is above the observed slope, we conclude that the slope is indeed different from 1 and that there is density dependence.

## Results

**Figure 3:**
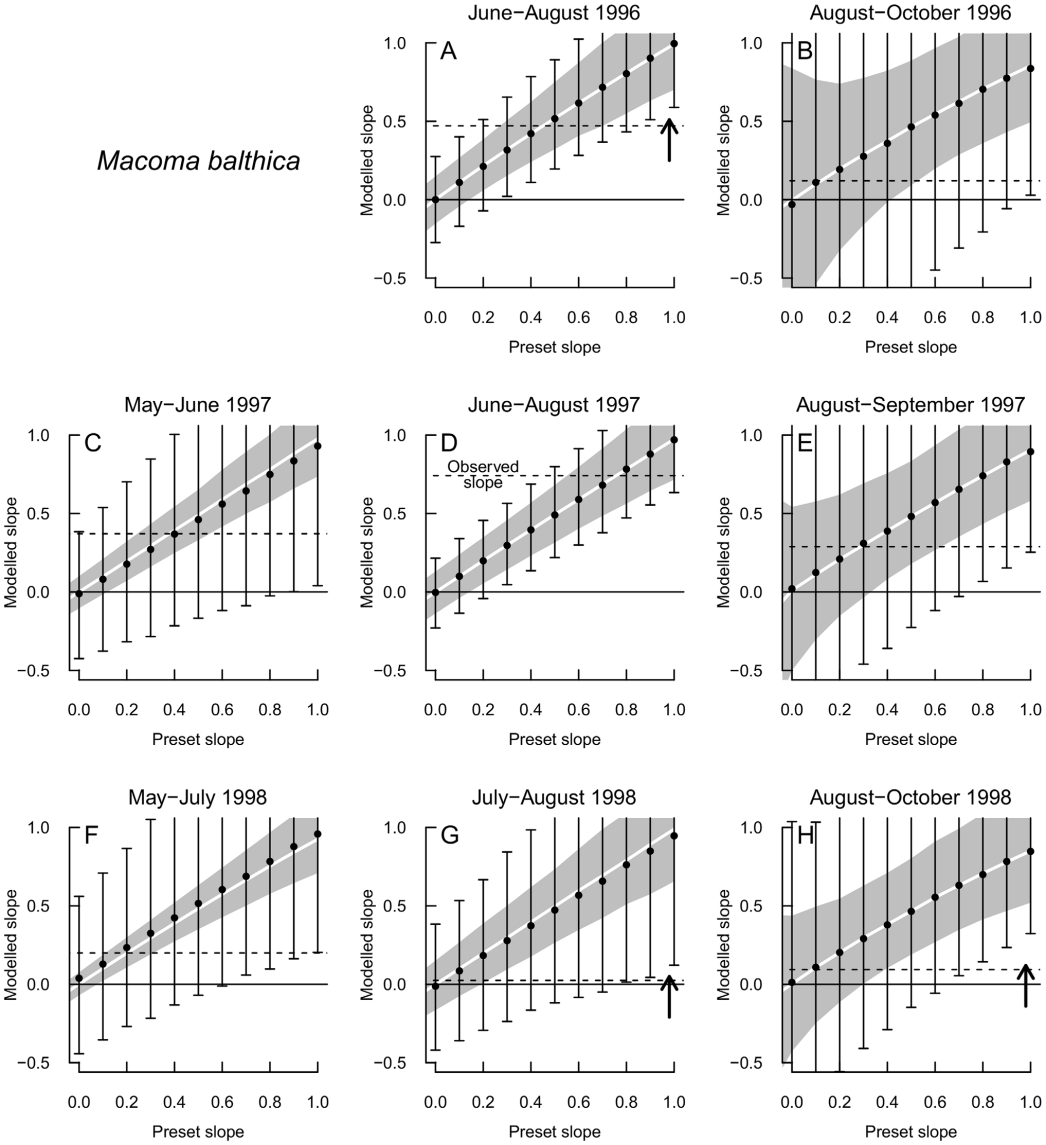
Simulation results for *Macoma balthica* in the time intervals of density decline. Dashed line: slope value of the original regression through the observed data. White line: average slope values resulting when measurement error is added to deterministic data on preset regression lines. Grey area: corresponding 95% confidence intervals. Black dots: average slope values resulting when process error and measurement error are added to deterministic data on preset regression lines. Whiskers: corresponding 95% confidence intervals. When the lower end of the confidence interval for the preset slope 1 ends above the observed slope value, the observed slope differs significantly from 1 and density dependence is concluded (arrows).

**Figure 4:**
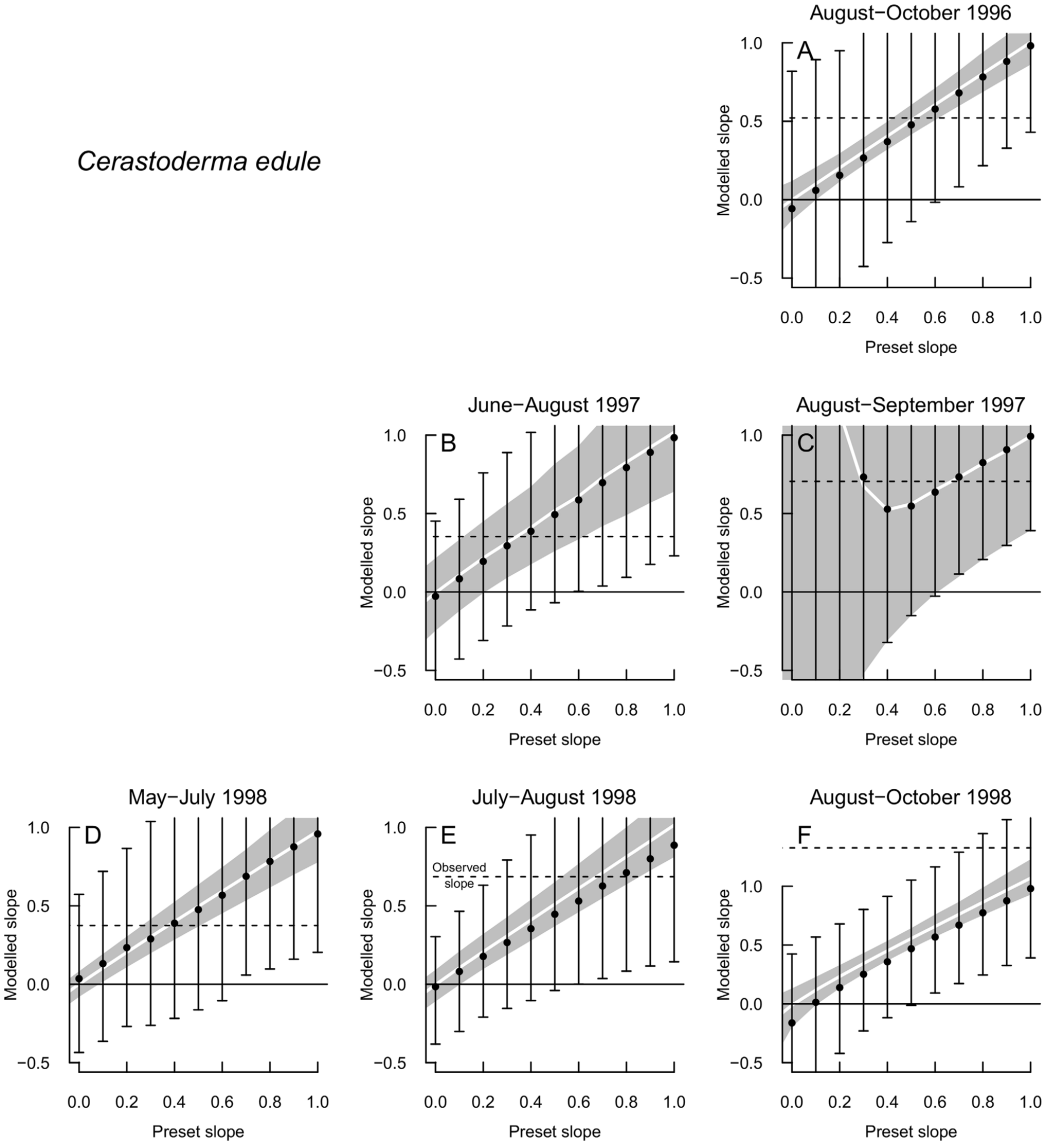
Simulation results for *Cerastoderma edule* in the time intervals of density decline. Dashed line: slope value of the original regression through the observed data. White line: average slope values resulting when measurement error is added to deterministic data on preset regression lines. grey area: corresponding 95% confidence intervals. Black dots: average slope values resulting when process error and measurement error are added to deterministic data on preset regression lines. Whiskers: corresponding 95% confidence intervals. If the lower end of the confidence interval for the preset slope 1 would end above the observed slope value, the observed slope would differ significantly from 1 and density dependence would be concluded.

**Figure 5:**
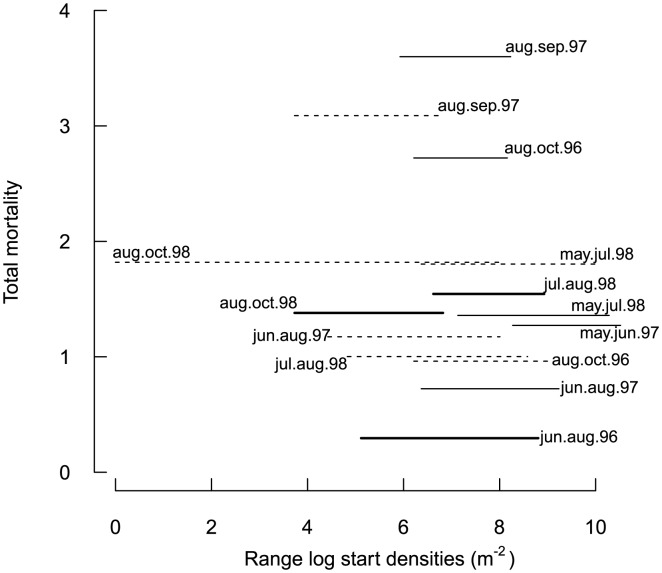
Mortality in relation to initial density. Range of log densities per m^2^ at the beginning of a time interval, and total mortality (mean log*D_t_* - mean log*D_t_*_+1_) during the time interval. Solid line: *Macoma balthica* (bold: density dependence detected). Broken line: *Cerastoderma edule*.

Three incidences of density dependence out of eight investigated cases were found for *Macoma balthica*, one of them in the middle of the summer 1996 and another two in 1998 in the time intervals in the middle and at the end of the summer (Fig. 3 A, G and H). Density dependence was concluded because the lower end of the modelled confidence interval for the preset slope 1 (no density dependence) ends above the observed slope value (arrows in Fig. 3), that means the observed slope differs significantly from 1. In *Cerastoderma edule* this was never the case (Fig. 4). The time intervals with density-dependent mortality did not start with an especially high density or large range of densities, nor were the mortalities exceptional (Fig. 5).

Adding measurement error to deterministic data of a given strength of density dependence generally lowered the average slope of regressions of log*D_t_*_+1_ on log*D_t_* (white lines in Fig. 3 and 4). The slope could take on a range of values in simulations with random error added (grey area in Fig. 3 and 4). The residual deviances of the regressions including measurement error were much lower than those of the original regressions through the observed data. Thus, the remaining variability must have been due to process variability. After extra error was added to log*D_t_*_+1_ to reach the original residual deviance, the average modelled slope changed only slightly compared to the slope with measurement error only (black points compared to white lines in Fig. 3 and 4), which is expected when error is added only to the y-variable and not to the x-variable. However, the range of possible slope values increased enormously (whiskers).

Concerning the strength of density dependence, in the first period where density dependence was observed for *M. balthica*, the preset slope that best predicted the observed slope when errors are included, was at about 0.45. By taking the logarithm and changing sign (i.e. reversing 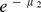 [15]), the density-dependent mortality coefficient is obtained: *μ*_2_ = 0.8. In practice this means for example that a 10-fold difference in initial abundances results only in a 2.8-fold difference in final abundances. The last two time intervals for *M. balthica*, for which density dependence was concluded, had slopes predicted near zero, which would mean that density-dependent mortality would almost cancel out all differences in initial densities. However, the estimates are not very precise. The range of slope values that can result from the simulations goes up as high as around 0.8, or a *μ*_2_ around 0.2. To illustrate this, a 4-fold difference in initial densities, as was found for example between peak total densities of the three years [20], would then still lead to a 3-fold difference in final densities. A 20-fold difference, as could be found between initial densities in space, would lead to an 11-fold difference in the end of the time interval. In the other investigated periods for *M. balthica*, and for *C. edule*, there was no evidence of density-dependent mortality, but with the large spread of possible results, it cannot be excluded either. The graph for *C. edule* in the last period of 1997 has matching measurement error and process error prediction intervals, this is because the measurement error estimated by bootstrapping was small, but we did not simulate underdispersed data. Without process error the residual deviance was higher than in the original regression in this case.

## Discussion

Density-dependent decrease was detected for pre-recruit *Macoma balthica* in three of eight investigated time periods, but there was no evidence for density dependence in *Cerastoderma edule*. Stronger regulation in *M. balthica* than in *C. edule* is expected from the higher variability of *C. edule* abundances [7]. While this gives confidence that the findings are due to actual processes, first we discuss the reliability of the results in relation to methodological aspects.

A few methodological drawbacks could have affected the results. An underestimation of the measurement error could have promoted the detection of density dependence (type I error). Within a transect, the choice of the positions of the sampling sites at the three tidal levels might have been an additional source of variation, that could not be quantified. As a consequence, the average resulting slope could have been too high, leading to false positive results. Also movements of animals between habitats can lead to population change appearing density-dependent [16]. In the first interval of abundance decrease in 1996, where density dependence was concluded for *M. balthica*, indeed an increase in density occurred in the two northernmost transects, pointing to a possible additional transport alongshore into the area that could have led to the significant result. However, in both bivalve species density-independent mortality dominated a majority of the time frames examined. Are there issues through which density dependence could have been missed? In *C. edule*, the occurrence of planktonic larvae is spread over a longer time [23], and smaller numbers of individuals might have settled into the area later. Although this had not led to a detectable increase of pre-recruits, it could have affected the perceived mortality for *C. edule*. Further, the process errors were generally large, which led to a large uncertainty of the estimates of the density-dependent mortality coefficient. In some cases only extremely strong density dependence would have been detectable (esp. Fig. 3B and C). Thus, occurrences of weak density dependence may have been overlooked due to a lack of power (type II error). A sampling round lasted several days, adding more apparent “process” variation, which could have contributed to the problem. From the population perspective, one has to bear in mind that we solely analyzed spatial density dependence, while temporal density dependence is more important for population dynamics [24]. To predict temporal density dependence from spatial density dependence one has to know the underlying biological mechanism [24]. Several density-dependent mechanisms can have an effect in space and in time. However, if predators react in aggregative response to relative density at a single point in time and not to absolute density, this is not necessarily a stabilizing process [25]. Limits to generalizations from this study are also set by its spatial and temporal extent.

Besides stating the presence or absence of density-dependent mortality, can we say more about the possible causes? That the process error is big is not only to be seen as something that reduces power, but, unlike the problems arising from inaccurate data, also has an ecological meaning. Most of the time, apparently stochastic processes dominated over density-dependent mortality. We did not investigate mechanisms directly, but anticipated getting indications for underlying processes from similarities or differences between species and time steps. In August-September 1997 the highest mortalities in *M. balthica* as well as in *C. edule* were measured (Fig. 5); both species probably suffered a strong common density-independent mortality source, such as physical disturbance. The data collection presented here accompanied predator exclosure experiments near one of the transects [26]. There is no support for predators causing density-dependent mortality in this study. No predation had been detected with the exclosure experiment in 1996, when predator arrival after the cold winter was delayed [23]. Predation on *M. balthica* and *C. edule* did occur in August 1997 and June 1998. These months do not match the periods with density-dependent mortality in *M. balthica* (although 3E and F appear nearly density-dependent). While with the smaller spatial extent, the exclosure experiment cannot be compared directly with this study, it suggests that predation might distort the effects of density-dependent mortality, possibly through variable predator access between sites. In contrast, van der Veer et al. [27] attributed density-dependent mortality in juvenile flatfish in the Wadden Sea to generalist crustacean predators shifting their diet to abundant prey, and 1996 with late crustacean arrival broke free of that relationship.

A potentially informative difference between the two species is in feeding mode and related consequences for competition and adult-juvenile interactions. Competition for food has been found stronger in deposit- than in suspension-feeding species [28], [29], leading to more constant population densities in deposit feeders and higher variability in suspension feeders [30] such as *C. edule*. Competition has not received much attention in the soft bottom intertidal, because it is not thought to be especially important, even less so in millimeter sized juveniles. Planktonic chlorophyll a levels, as a proxy for primary production for which clams could have competed, were not highest in 1997 [31], the year with no density dependence detected in *M. balthica*. There are examples of a negative influence of high adult abundances on juveniles [32]. Adult-juvenile interactions have been proposed as a reason for the horizontal stock-recruitment relationship in *C. edule* [33], but their adults should affect juveniles of other species as well. We investigated and found evidence for spatial density-dependence among juvenile *M. balthica*, but in this species adult and juvenile distributions are not even correlated, because of an ontogenetic habitat shift. Habitat choice might be a relevant issue for explaining density independence of bivalve settler mortality. High density may be correlated with good survival as secondary settlers choose sites with high quality, and this overrules any spatially density-dependent mortality.

Much of the literature and debate on density dependence is about annual population changes. Fewer studies are concerned with density dependence within a single cohort (e.g. [22], [34], [35]), and several different approaches are used. Density dependence within cohorts in time intervals shorter than a year has hardly been addressed. This study is the first to investigate density dependence within the early months of the life of Wadden Sea bivalves at a regional scale, on the basis of a well-defined statistical model, and assessing the effects of measurement and process error on the precision of parameter estimates by means of simulation studies. Our analysis method can be used for other systems as well, and for other data arrangements that do not confound density with age [15]. We found evidence for both density-dependent and independent mortality, but density independence was more common. This is a reminder that, although density dependence is expected from the stock-recruitment relationship, also the enormous variation around the horizontal part of the curve should be acknowledged. Previous studies on bivalve recruitment [36]–[38] have been on an important track by taking account of density-independent influences. Understanding recruitment remains difficult, as hardly any factors can be dependably predicted. Knowledge of the processes that shape survival until recruitment is important, because recruitment is the key to population dynamics of mass spawners. In recent years *M. balthica* abundances in the Western Wadden Sea strongly decreased [39] and now at low stock a stock-recruitment relationship has become apparent ([40] Fig. 7.2). One should remember that density-dependent mortality does not necessarily mean that populations have the ability to recover when they are reduced to low numbers [41]. Even if subsequent survival is expected to be better at low density, through an Allee effect [42] in these broadcast spawners, ultimately reproductive success will decrease at low adult density, as fertilization fails through dilution of gametes [43]. It is not known whether the recent decrease in abundances of both adult and juvenile *Macoma balthica* is an internal dynamic, or if they suffer from an extrinsic mortality source they have in common.

In this observational study, comparisons between species and time periods did not shed enough light on the processes behind density-dependent mortality. In future research, the mechanisms should be studied directly. Density-dependent infections with pathogens are becoming a strong candidate and have hardly been studied. Further, the theoretical consequences of density-dependence at different times in the life cycle on population dynamics could be investigated.
